# Commentary on the article: Are hepatitis B virus and celiac disease linked? HBV and celiac disease

**Published:** 2011-01-01

**Authors:** Fabiana Zingone, Carolina Ciacci

**Affiliations:** 1 Gastroenterology, Department of Clinical and Experimental Medicine, Via Sergio Pansini, Naples, Italy

**Keywords:** Celiac, Hepatitis B virus

Dear Editor,

The recent paper from Leonardi and La Rosa (2010) aimed to establish the prevalence of celiac disease (CD) in patients with chronic hepatitis B (HBV) infection [1]. Previous studies suggest that chronic hepatitis C (HCV) could trigger immunological gluten intolerance in susceptible patients [2]. This hypothesis, however, is not yet supported by sufficient scientific evidence. Similarly, the possible induction of CD in patients treated with interferon (IFN) therapy is supported in literature only by case reports [3]. Moreover, CD affects as much as 1% of the general population [4], and it is possible that the association described in some papers is the result of incidental findings in a population most studied for the underlying disease. Although the study by Leonardi and La Rosa (2010) was unable to disclose any association between CD and HBV infection, it represents an interesting starting point. The study does have some limitations, particularly a small sample size, a very young average age of participants, and an overrepresentation of men in the sample. In fact, a typical population affected with CD is mostly made up of young adults, with twice as many women as men. In this more typical population, a diagnosis of CD is made according to the presence of EMA and antitransglutaminase IgA in the blood and then an intestinal biopsy. The relationship between CD and hepatitis viruses is intriguing. Previous studies have indicated a defective response to the hepatitis B vaccine in CD. It has been postulated that a primary role of human leukocyte antigen (HLA) genotype DQ2 is that it is predisposed to low immunity to recombinant hepatitis B vaccine [5]. Other studies have postulated a possible role of gluten intake at the time of vaccination [6]. Therefore a larger study should be designed to investigate the prevalence of CD in individuals with HBV and HCV and the possible activation of CD with IFN therapy. Such a study should also include an analysis of the HLA genotype, which seems to have a principal role in this association.
